# Case report: Fecal microbiota transplant for *Clostridium difficile* infection in a pregnant patient with acute severe ulcerative colitis

**DOI:** 10.3389/fimmu.2024.1417003

**Published:** 2024-11-21

**Authors:** Hanyu Wang, Feihong Deng, Min Luo, Xuehong Wang

**Affiliations:** ^1^ Department of Gastroenterology, The Second Xiangya Hospital, Central South University, Changsha, Hunan, China; ^2^ Research Center of Digestive Disease, Central South University, Changsha, Hunan, China

**Keywords:** acute severe ulcerative colitis, fecal microbiota transplant, *Clostridium difficile* infection, pregnancy, gut microbiota

## Abstract

Ulcerative colitis (UC) is a chronic colonic mucosal inflammation characterized by reduced gut microbial diversity. Patients with UC at pregnancy are prone to suffer from severe disease progression due to the changes of hormone and immune regulation. Fecal microbiota transplant (FMT) is a promising therapy for UC and recurrent *Clostridium difficile* infection (CDI). However, acute severe ulcerative colitis (ASUC) treatment especially in patients at pregnancy is clinically challenging. Herein, we report a 34-year-old pregnant woman who manifested with numerous bloody stools and markedly elevated serological inflammatory indicators and was diagnosed with ASUC and concurrent CDI. The use of intravenous injection steroids and anti-TNF-α therapy failed to improve her condition. Frozen encapsulated FMT therapy was finally performed to this patient with clearly improved symptoms and indications of safe delivery without UC flares or complications, and markedly increased diversity of the gut microbiota was also shown in this patient after FMT. This report firstly describes FMT as a safe salvage therapy for a pregnant patient with CDI and ASUC refractory to intravenous steroids and anti-TNF therapy.

## Introduction

Ulcerative colitis (UC) is a chronic inflammatory bowel disease (IBD) characterized by continuous mucosal inflammation of the colon and rectum. It markedly affects young people during their reproductive period as the course of UC during pregnancy correlates with the level of the disease activity at the time of conception (1, 2). A severe flare of UC during pregnancy poses significant risks of adverse maternal and fetal outcome; therefore, appropriate management of UC during pregnancy is particularly crucial (2). Patients with severe UC are more likely to experience *Clostridium difficile* infection (CDI). Over the last decades, fecal microbiota transplant (FMT) has been recognized as an effective treatment for both UC and CDI (3, 4). There are limited data describing the safety of FMT therapy in pregnant patients and its efficacy for patients with UC with CDI. Here, we firstly report a case of successful FMT in a pregnant patient with acute severe UC and combined CDI, as well as an analysis of the microbiota of fecal samples obtained before and after FMT.

## Case report

A 34-year-old woman gravida 6/para 3 at 26 weeks’ gestation with a 1-year history of ulcerative pancolitis was admitted on 10 July 2022 due to a UC flare, which was characterized by increased stool frequency up to 10 times a day, bloody diarrhea, and cramping abdominal pain. In June 2021, she was firstly diagnosed with UC and was treated with mesalamine, which resolved the diarrhea and abdominal pain. The patient had self-discontinued mesalamine therapy during pregnancy without medical counseling. The patient’s present symptoms commenced 2 months prior to admission and worsened 10 days before hospitalization.

**Figure 1:**
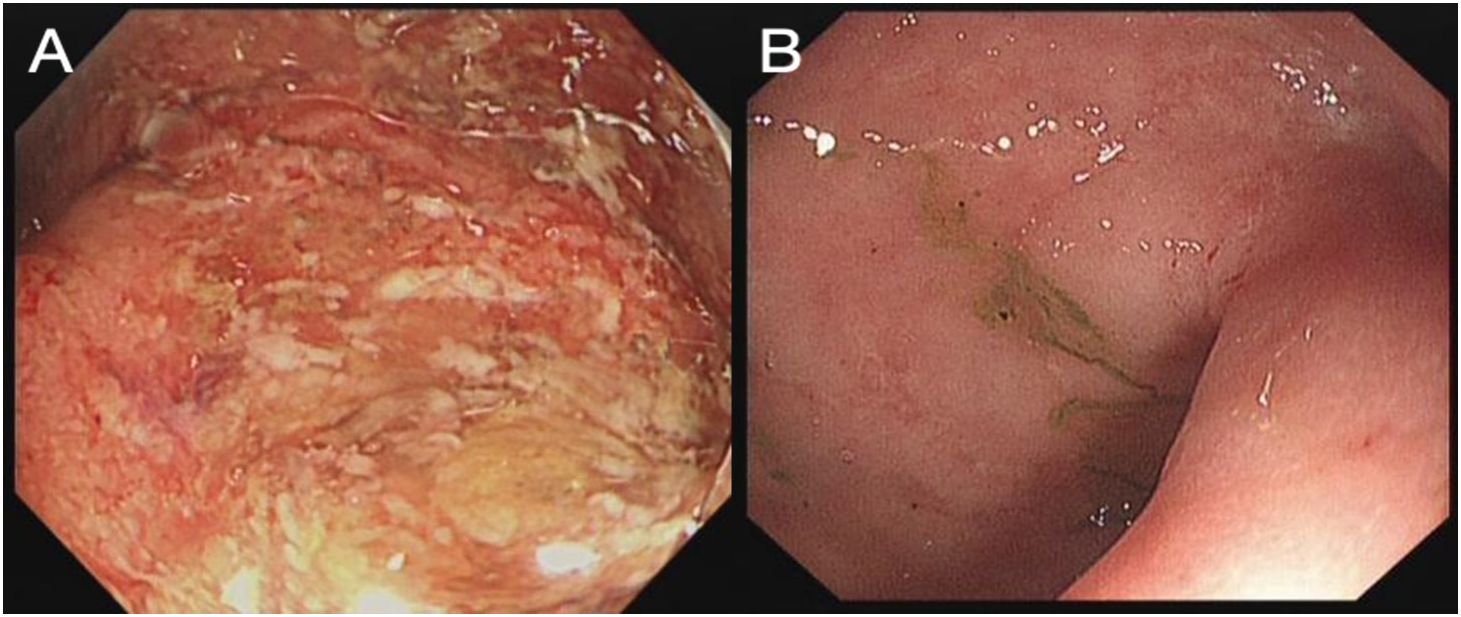
Rectum image of flexible sigmoidoscopy investigation before FMT **(A)** and 3 weeks after FMT **(B)**.

Upon admission, she was afebrile, with a pulse rate (PR) of 90 bpm and a blood pressure (BP) of 102/70 mmHg. Routine blood laboratory tests indicated mild anemia (Hb 109 g/L, normal range 115–135 g/L), hypoalbuminemia (32 g/L, normal range 40–55 g/L), and elevated C-reactive protein (CRP, 78 mg/L, normal range 0–5 mg/L) and erythrocyte sedimentation rate (ESR, 74 mm/h, normal range 0–20 mm/h), while cytomegalovirus (CMV) DNA was negative. Fecal culture was negative for standard pathogens; however, *C. difficile* toxins and polymerase chain reaction testing were positive. A sigmoidoscopy was performed by an experienced endoscopist, which revealed severe proctitis with an endoscopic Mayo score of 3 (**Figure 1A**). The biopsies obtained by sigmoidoscopy excluded cytomegalovirus infection. According to the modified criteria of Truelove and Witts (5), this patient was diagnosed with acute severe ulcerative colitis (ASUC) concurrent with CDI. She was then administered with intravenous (IV) methylprednisolone (40 mg per day) and oral vancomycin (125 mg, four times a day).

After 3 days of methylprednisolone treatment, the defecation frequency was still 9–10 times a day and she subsequently received infliximab (300 mg once) salvage therapy. Although she had some reduction of bowel frequency to seven to eight bowel actions per day in accordance with the decrease in severity of abdominal pain, she continued to have intermittent bloody diarrhea and constant levels of CRP (79 mg/L) and ESR (86 mm/h) 10 days later. Therefore, FMT was taken into consideration. The fresh healthy intestinal flora comes from the fecal bank and is stored in capsules at low temperature; the mean stool per capsule was 0.75 g. Donor material was prepared at a stool bank, and the patient received 15 g each time and three times a week. After signing the informed consent, she underwent FMT approved by the ethics committees of the Second Xiangya Hospital on 27 July 2022.

On day 4 after FMT, the patient reported significant relief of her abdominal pain and a reduction of bowel frequency to three times per day. On day 18 after FMT, the patient recovered well and had a normal bowel pattern, with CRP decreased to 1 mg/L. A flexible sigmoidoscopy was performed before discharge, which revealed a great improvement of the colonic mucosa (**Figure 1B**).

**Figure 2:**
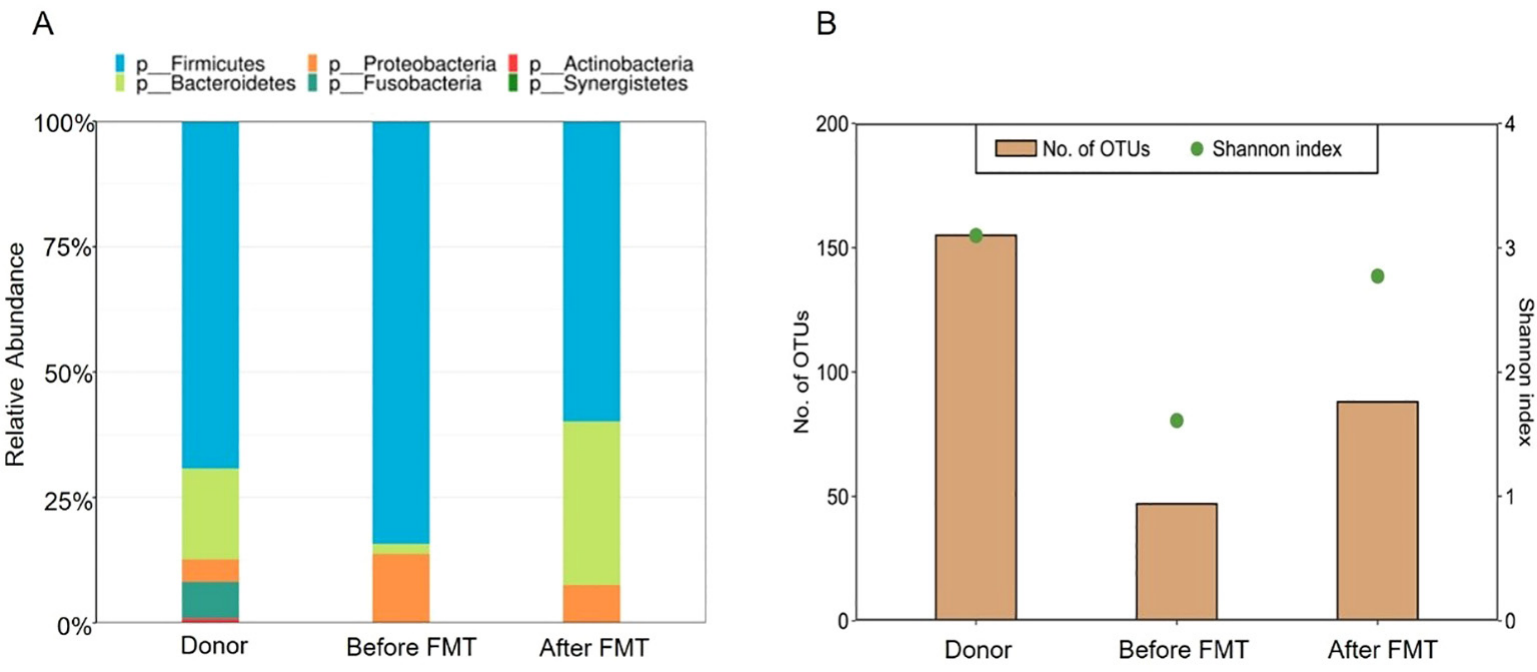
Composition and diversity of the gut microbiome. **(A)** Relative abundance of the donor and patient, before and after FMT. **(B)** Shannon index of microbiota diversity and total number of OTUs of the donor and patient, before and after FMT.

The patient was discharged from the hospital 3 days later. She continued to receive infliximab and mesalamine as maintenance therapy. The gut microbiota of the stool specimens obtained from the patient before FMT and 3 weeks after FMT were analyzed by 16S rRNA gene sequencing. Post-FMT samples from the patient showed an increase in the abundance of *Bacteroidetes*, accompanied by decreased abundance of *Proteobacteria* compared to that found in the patient before FMT (**Figure 2A**). Furthermore, the total number of operational taxonomic units (OTUs) before FMT was under 50, whereas the total number of OTUs after FMT was 85. Shannon index improved from 2.7 to approximately 3.8 after FMT (**Figure 2B**).

**Figure 3:**
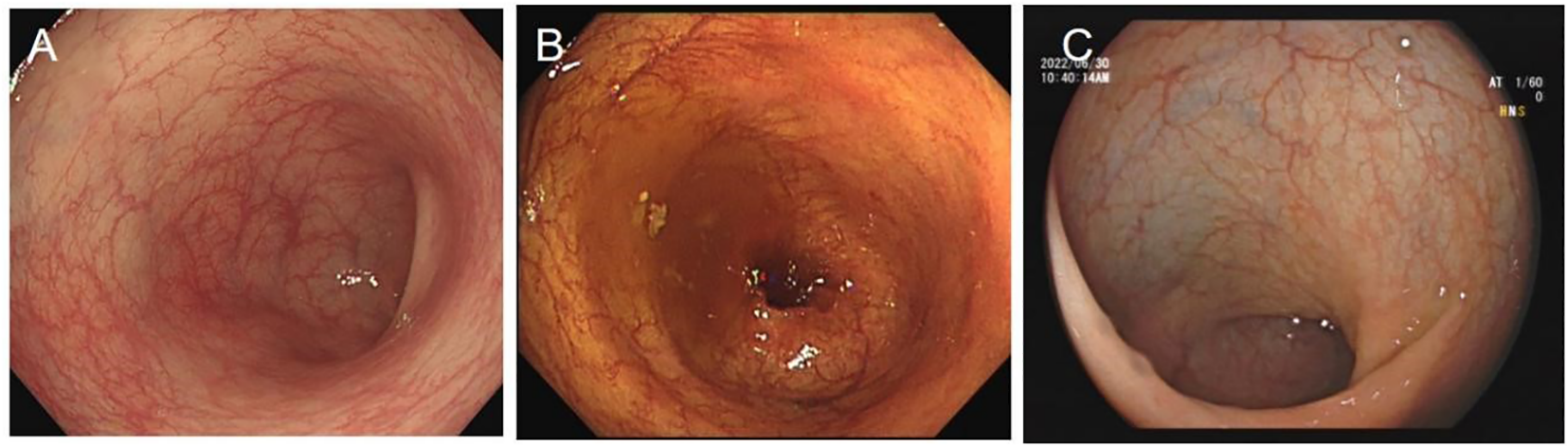
Rectum image of follow-up flexible sigmoidoscopy screening after FMT at **(A)** 5 months, **(B)** 10 months, and **(C)** 15 months.

She delivered at 37 + 3 weeks of gestation on September 23. Both the patient and the newborn were in good clinical condition (2.96 kg, APGAR 10/10). Follow-up after 5, 10, and 15 months of FMT revealed that the infant was developing normally, and the patient had no sign of recurrence along with endoscopic healing (**Figure 3**).

## Discussion

UC progression during pregnancy correlates with the activity at the onset of pregnancy (2, 6). Treatment of ASUC in combination with CDI is clinically challenging; FMT is believed to treat recurrent CDI with high efficacy by restoring the diversity of intestinal microbiota (7), whereas studies of FMT in ASUC treatment are limited. In this case, we firstly reported a pregnant patient who suffered from ASUC combined with CDI. FMT not only dramatically resolved the symptoms and facilitated safe delivery, but also markedly restored the diversity of the gut microbiota.

A decrease of gut-dominant microbiota causes intestinal microbiota imbalance, then pathogens or conditional pathogens invade the lamina propria layer to trigger mucosal inflammation and lead to disease such as IBD (8). IBD is typically characterized by reduced microbial diversity (8). For normal pregnancy, the overall diversity of the gut bacteria was also reduced; in the third trimester, a dysbiosis was observed, showing a state of low-grade inflammation of the gastrointestinal tract (9). Pinto Y et al. found that the gut microbiome appears to play a role in inflammation-induced gestational diabetes mellitus (GDM) pathogenesis (10). Additionally, because of the Western style diet during pregnancy, microbial changes are augmented especially in patients with IBD. Mahadevan et al. reported that higher disease activity was associated with risk of spontaneous abortion (HR 3.41, 95% CI 1.51–7.69) and preterm birth with increased infant infection (OR 1.73, 95% CI 1.19–2.51) in pregnant women with IBD (11). Additionally, patients with UC had significantly increased disease activity compared to CD during each trimester (11). In particular, pregnancy causes immunological changes at the fetal/maternal interface, presenting with a predominantly Th2 phenotype (9). It can be speculated that Th1-mediated diseases such as CD may benefit from pregnancy-induced changes, while Th2-mediated diseases such as UC might be aggravated, revealing that pregnant women at their third trimester combined with active UC might have worsened disease progression.

The clinical guidelines of the management of IBD in pregnancy have recommended that pregnant women with IBD who have a disease flare on optimal 5-ASA should be treated with systemic corticosteroids or anti-TNF therapy to induce symptomatic remission (2). Patients will need salvage therapy if they do not respond to 3 days of IV steroid treatment based on the Oxford index (12), and anti-TNF therapy is generally recommended (2, 13). In this case, corticosteroid treatment failed to improve the patient’s condition, and the patient subsequently received infliximab therapy to control disease activity. The patient was diagnosed with ASUC and concurrent CDI, and her symptoms were not apparently relieved after 10 days of infliximab treatment. Previously, a 28-year-old pregnant woman who presented with recurrent CDI received good outcome after FMT (14). Because of the good medical condition of the premature fetus in the womb of this pregnant patient, FMT is a better treatment option for her than surgical colectomy.

FMT has been previously reported to be effective for recurrent CDI with low adverse event profile and high efficacy rates (4). FMT is also an effective and safe strategy to induce long-term remission in patients with active UC. Fang et al. reported the long-term efficacy and safety of monotherapy with a single fresh FMT for recurrent active UC. The median remission time was 24 months (95% CI 68.26%–131.7%) in the FMT group (range 6–38 months) (15). No side effects were observed, and no infection with certain pathogens was observed during long-term follow-up in this study. Feng et al. conducted a search and compiled 13 randomized controlled trials (RCTs) that were of high quality and focused on the use of FMT for UC treatment (16). They found that the FMT group demonstrated significantly improved rates of both clinical remission and endoscopic remission when compared to the control group. FMT can regulate gut immune system function by affecting the differentiation of T cells, reduce intestinal permeability, and thus influence the occurrence and development of intestinal inflammation (17). Singh et al. found that early FMT after donor defecation favorably impacts the clinical response rates in patients with active UC (18). The mode of FMT delivery is also critical for drug efficacy. Nanki et al. have performed FMT via colonoscopy to treat an 82-year-old woman with recurrent CDI (19). Entire colonoscopy increased the risks for a pregnant patient at her third trimester. Crothers et al. reported that oral frozen encapsulated FMT is a promising FMT delivery system and may be preferred for long-term treatment strategies in UC and other chronic diseases (20). In this case, we firstly reported a pregnant patient diagnosed with ASUC combined with CDI who received frozen encapsulated FMT treatment and obtained clinical and endoscopic remission with high efficacy; additionally, after FMT, the diversity of the intestinal microbiota was increased, showing the increased abundance of *Bacteroidetes* and decreased abundance of *Proteobacteria* and *Fusobacterium*. Furthermore, this patient experienced a UC long-term clinical and endoscopic remission (15 months) without adverse events, accompanied by the healthy growth of the infant.

In summary, we firstly reported that FMT was a safe salvage therapy for a pregnant patient with ASUC and CDI who failed to respond to IV steroids and anti-TNF therapy. The frozen encapsulated FMT is a promising FMT delivery system that is convenient and with high efficacy. Further larger trials should be carried out to explore the benefits of frozen encapsulated FMT and ASUC combined with CDI.

## Data Availability

The data presented in the study are deposited in the Sequence 7 Read Archive repository, accession number PRJNA1145901.
